# Exploration of the impact of air pollutants on the influenza epidemic after the emergence of COVID-19: based on Jiangsu Province, China (2020–2024)

**DOI:** 10.3389/fpubh.2025.1555430

**Published:** 2025-04-14

**Authors:** Chengxi Zheng, Xin Jiang, Yi Yin, Qigang Dai, Shuhan Tang, Jianli Hu, Changjun Bao, Haitao Yang, Zhihang Peng

**Affiliations:** ^1^School of Public Health, Nanjing Medical University, Nanjing, China; ^2^Department of Acute Infectious Diseases Control and Prevention, Jiangsu Provincial Center for Disease Control and Prevention, Nanjing, China; ^3^National Key Laboratory of Intelligent Tracking and Forecasting for Infectious Diseases, Chinese Center for Disease Control and Prevention, Beijing, China; ^4^Chinese Center for Disease Control and Prevention, Beijing, China

**Keywords:** influenza, air pollutants, age-specific transmissibility, DLNM, COVID-19

## Abstract

**Background:**

Non-pharmaceutical interventions (NPIs) during the COVID-19 pandemic altered influenza transmission patterns, yet the age-specific effects of air pollutants on influenza dynamics remain unclear.

**Methods:**

Utilizing influenza surveillance data of Jiangsu Province from 2020 to 2024, we integrated generalized additive quasi-Poisson regression model and distributed lag non-linear models (DLNM) to quantify lagged effects and exposure-response relationships between air pollutants (NO_2_, SO_2_, PM_2.5_) and influenza risk across young, middle-aged, and older adult groups. Meteorological factors, including temperature and humidity, as well as the implementation stages of NPIs, were controlled in the model to isolate the impact of pollutants on influenza transmission.

**Results:**

The NO_2_ and SO_2_ both showed significant positive effects in all age groups. The effect of NO_2_ is most significant in the young group (RR = 5.02, 95% CI: 4.69–5.37), while SO_2_ exhibited the most pronounced effects in middle-aged and older adult groups (RR = 4.22, 95% CI: 3.36–5.30; RR = 8.31, 95% CI: 5.77–11.96, respectively). PM_2.5_ elevated risks in young (RR = 1.99, 95% CI: 1.87–2.12) and older adult (RR = 1.45, 95% CI: 1.07–1.94) groups. Interactions between meteorological factors (temperature, humidity) and pollutants were statistically insignificant.

**Conclusions:**

Air pollutant impacts on influenza transmission are age-dependent: NO_2_ dominates in younger populations, whereas SO_2_ disproportionately affects older adults. These findings highlight age-related vulnerability to air pollution and the need for targeted public health strategies for different population subgroups.

## 1 Introduction

Influenza, an acute respiratory illness caused by the influenza virus, poses a significant threat to public health worldwide. Despite the availability of vaccines and antiviral medications, the transmission rate of influenza remains high in the winter due to temperature fluctuations (1). Environmental factors, particularly air pollutants and meteorology, have been increasingly highlighted in the transmission of influenza. Through a synergistic effect, environmental factors pose spatio-temporal effects on influenza dynamics (2).

Meteorological factors, such as temperature and humidity, are crucial determinants of influenza transmission (3). Survival and transmission characteristics of influenza viruses distinctly differ with climatic conditions (4). Low temperature and humidity, more encountered in winter, create an environment that is conducive to viral transmission, while warm temperature and high humidity usually keep respiratory viruses stable (5).

Air pollutants, especially fine particulate matter and nitrogen oxides (NO_2_), have been established to exert adverse effects (6, 7). In addition to direct health impacts on respiratory, cardiovascular and immune systems, air pollutants indirectly elevate the susceptibility to infectious diseases through providing a favorable environment for the survival and spread of viruses, indicating its critical role in the transmission chain (8–10). For example, air pollutants maintain the stability of influenza viruses in the atmosphere, thereby increasing the likelihood of transmission over greater distances (11). In an age of industrialization, air pollution has become a severe issue in China (12). Excessive PM_2.5_ in the respiratory system damages the immune function, thus increase the risk of contracting influenza (13). In heavily polluted regions, air pollutants enhance the viability of airborne viruses and thus prolong the survival and facilitate the transmission of viruses (9, 10). In addition, air pollution may indirectly enhance the spread of the virus by compromising respiratory functions and disrupting immune responses (14). The impact of air pollution on influenza varies among different age groups, with weaker immune systems such as children and the older adult being more susceptible to the effects of air pollution, thereby increasing the risk of contracting influenza (15, 16).

The COVID-19 pandemic has introduced new complexities into the dynamics of influenza transmission. Non-pharmaceutical interventions (NPIs) implemented during the pandemic, such as mask-wearing and social distancing, initially led to a significant reduction in influenza activity globally (17). However, as these measures were relaxed, influenza activity rebounded, highlighting the delicate balance between viral suppression and resurgence (18).

In the present study, we analyzed the potential impacts of air pollutants on influenza in different age groups. Through quantifying their influences, our findings are expected to provide evidence-based insights into the age-specific effects of air pollutants on influenza transmission, enabling the development of targeted prevention and treatment strategies tailored to vulnerable populations, particularly in the context of COVID-19.

## 2 Materials and methods

### 2.1 Design overview

This study analyzed influenza surveillance data, air pollution and meteorological data during the implementation of non-pharmaceutical interventions (NPIs) during the COVID-19 pandemic in China. The onset of the pandemic was used as the starting point, and key time points when significant changes in China's policies were identified. Study period started from the 1st week of 2020 and ended at the 17th week of 2024, and divided into two stages: from January 1st, 2020 to December 26, 2022; from December 27, 2022 to April 22, 2024. The prevalence of influenza at different time points of NPIs implementation, and the relationship of air pollutants and influenza was examined. The cohort was divided into three groups of young, middle-aged, and older adults.

### 2.2 Influenza data and two NPIs stages

ILI was defined as fever (temperature ≥38°C) accompanied by cough or sore throat symptoms. According to the National Influenza Monitoring Program requirements, influenza data are monitored by medical staff at sentinel hospitals (in internal medicine outpatient departments, internal medicine emergency departments, fever clinics, pediatric outpatient departments, and pediatric emergency departments). The number of ILI cases and the total number of outpatient visits were reported daily in the three age groups, and put into the China Influenza Monitoring Information System at specified times. The ILI percentage was defined as the ratio of ILI cases reported weekly to the total number of outpatient visits for that week.

With support from the Major National Science and Technology Projects for the Prevention and Control of Infectious Diseases, China has established a national influenza monitoring network. Currently, the network comprises 411 laboratories and 556 sentinel hospitals. We collected influenza data weekly reported by sentinel hospitals in Jiangsu Province from the 1st week of 2020 to the 17th week of 2024, including the number of ILI cases and the total number of outpatient visits in the three age groups. The data were classified and summarized based on city codes and station codes. Regarding quality control, the data collected from sentinel hospitals underwent strict standardization and validation procedures. All data were cross-checked and cleaned multiple times to ensure accuracy and consistency.

This study period was divided into two stages. The first stage was from December 30th, 2019 to December 26, 2022, during which COVID-19 gradually became pandemic in China and NPIs were implemented, such as home isolation, city lockdowns, and mask-wearing. The second stage was from December 26, 2022 to April 22, 2024, during which NPIs were gradually loosened and lifted in China.

### 2.3 Patient and public involvement

Patients or the public were not involved in the design, or conduct, or reporting, or dissemination of our research.

### 2.4 Meteorological and air pollution data

Data of major air pollutants were obtained from the China National Environmental Monitoring Center (http://106.37.208.233:20035/), including NO_2_ (μg/m^3^), sulfur dioxide (SO_2_) (μg/m^3^), and particulate matter <2.5 μm (PM_2.5_) (μg/m^3^). Meteorological data in Jiangsu were obtained from National Meteorological Information Center, including average temperature (°C) and relative humidity (%) with a resolution of 0.25° × 0.25° (http://data.cma.cn/).

### 2.5 Statistical analysis

The cohort was divided into five age groups (0–5 years, 5–15 years, 15–25 years, 25–60 years, and over 60 years). We further combined the five age groups into three groups, 0–25 (young group), 25–60 (middle-aged group), and over 60 (older adult group).

The generalized additive quasi-Poisson regression model was combined with the distribution lag non-linear model (DLNM) to explore the relationships between the air pollutants and the number of ILI cases in the three age groups. A “cross-basis” matrix was constructed for each air pollutant, accounting for both the exposure-response relationship and lag effects. The exposure-response relationship was described using a linear function, while the lag effects were modeled using basic spline function. The formula of the combined model is:


(1) $$\begin{array}{r} \log\left[E\left(Y_{t}\right)\right]=a+cb\left(x_{i},lag,df\right)+\sum ns\left(x_{j},df\right)\\ +ns\left(time,df\times 6\right)+\delta \left(week\right)+\delta \left(stage\right) \end{array}$$

where *Y*_*t*_ represents the weekly number of influenza cases on week t in an age group; a is the intercept; cb represents the cross-basis matrix of air pollutants; ns() denotes a natural cubic spline function; *x*_*i*_ represents one air pollutant, such as NO_2_ (μg/m^3^), SO_2_ (μg/m^3^) and PM_2.5_ (μg/m^3^); *x*_*j*_ represents the air pollutants other than *x*_*i*_, as well as average temperature and relative humidity; time signifies long-term trends and seasonality; week indicates the number of the week; stage refers to the onset of the COVID-19 pandemic and the timeline of NPIs in China; and df represents the degrees of freedom. Based on the minimum Akaike information criterion (AIC), we chose the combination of the optimal parameters, including df = 3 for *x*_*j*_; df = 7 for time; and df = 4 is used to fit the nonlinear effect of the lags. A lag time of 4 weeks was chosen for meteorological factors based on the AIC (details are shown in the Supplementary material, Supplementary Tables S1, S2). In the formula, when studying the association between pollutants and influenza, we are comparing based on the pollutant concentration where the relative risk of influenza is at its minimum.

Finally, we explored the interaction between temperature and relative humidity on the risk of influenza based on a generalized additive model (GAM). The model is expressed as:


(2) $$\begin{array}{l} \log\left[E\left(Y_{t}\right)\right]=\beta_{1}+s_{1}\left(k,x\right)+strata \end{array}$$

β_1_ denotes intercept; represents a meteorological factor (average temperature or the relative humidity); *x* represents the concentration of an air pollutant; and *s*_1_(*k, x*) denotes the interaction between variables *k* and *x*.

The effect of the interaction between average temperature, relative humidity and air pollutant concentrations on influenza was quantitatively explored. With the median as the standard, the meteorological factors were divided into “low” and “high”. All combinations are calculated and compared to obtain RR values, relative excess risk due to interaction (RERI), attributable proportion due to interaction (AP) and synergy index (SI).

Given the significant effects of extreme pollutants change on influenza, we further quantified the impact of pollutants at the 99th percentile on influenza compared with the that at the 1st percentile. The RR values of influenza were calculated to quantify the impact of each air pollutant. All statistical analyses were performed using statistical software R (version 4.3.0).

## 3 Results

From December 30th, 2019 to April 22, 2024, the total number of outpatient and emergency visits reached 72,064,406, and the number of ILI cases reached 3,941,923, accounting for 5.47% of all visits. Since 2020, the proportion of ILI cases had decreased to ~5%. Upon the lifting of NPIs in the beginning of 2023, the proportion of ILI cases in the total number of outpatient and emergency department visits increased to 8.14%.

Supplementary Figure S1 illustrates the ILI cases across the three age groups, with the largest number in the young adult group. The changing trends in case numbers were generally similar across the three groups, with a peak commonly observed at the end of 2022. From 2020 to the end of 2022, the influenza epidemic was stable. In the beginning of 2023, the number of ILI cases, including some COVID-19 patients, surged.

Over the study period, the levels of air pollutants exhibited different trends. The air concentrations of PM_2.5_ and NO_2_ fluctuated. Meanwhile, the SO_2_ level is relatively low and stable. The average temperature exhibits seasonal fluctuations, while the relative humidity remains relatively stable (Table 1 and Supplementary Figure S2).

**Table 1:** Descriptive analysis of the data about influenza epidemics, air pollutants, and meteorological factors.

**Variables**	**Mean ±SD**	**Min**	**Max**	**P50(P25, P75)**
**Influenza epidemics**				
ILI cases	17,520.00 ± 17,798.40	2,610	102,905	10,941 (6,826, 21,878)
**Air pollutants**				
PM_2.5_ (μg/m^3^)	35.34 ± 16.78	11.92	94.54	31.31 (23.54,42.00)
SO_2_ (μg/m^3^)	7.40 ± 1.40	4.92	13.39	7.15 (6.39,8.08)
NO_2_ (μg/m^3^)	27.83 ± 11.10	10.38	70	25.46 (19.08,34.54)
**Meteorological factors**				
Average temperature (°C)	16.18 ± 9.05	−2.35	33.12	16.25 (8.14, 24.49)
Relative humidity (%)	73.50 ± 9.15	49.69	90.85	74.23 (67.38, 80.85)

**Figure 1:**
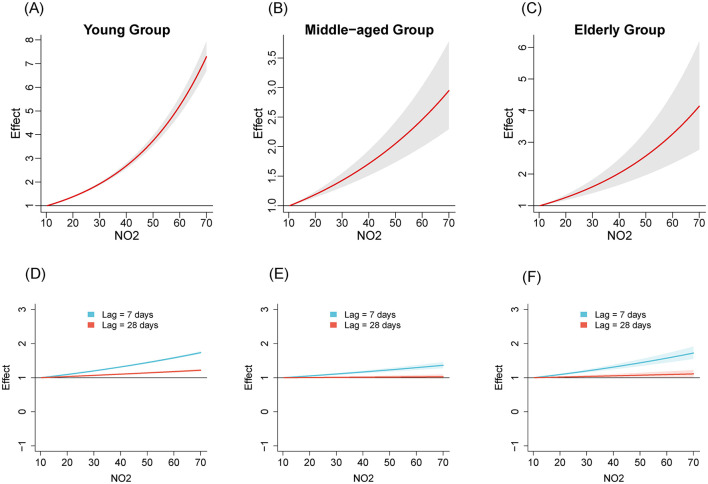
The estimated overall effects of NO_2_ (μg/m^3^) on influenza across 3 age groups using DLNM. Top row **(A–C)**: Cumulative effects of NO_2_ over the entire lag period, representing the overall impact on influenza risk. Bottom row **(D–F)**: Specific risk of influenza at lag periods of 7 days (**blue line**) and 28 days (**red line**). **(A, D)** Young group, **(B, E)** middle-aged group, **(C, F)** older adult group.

**Figure 2:**
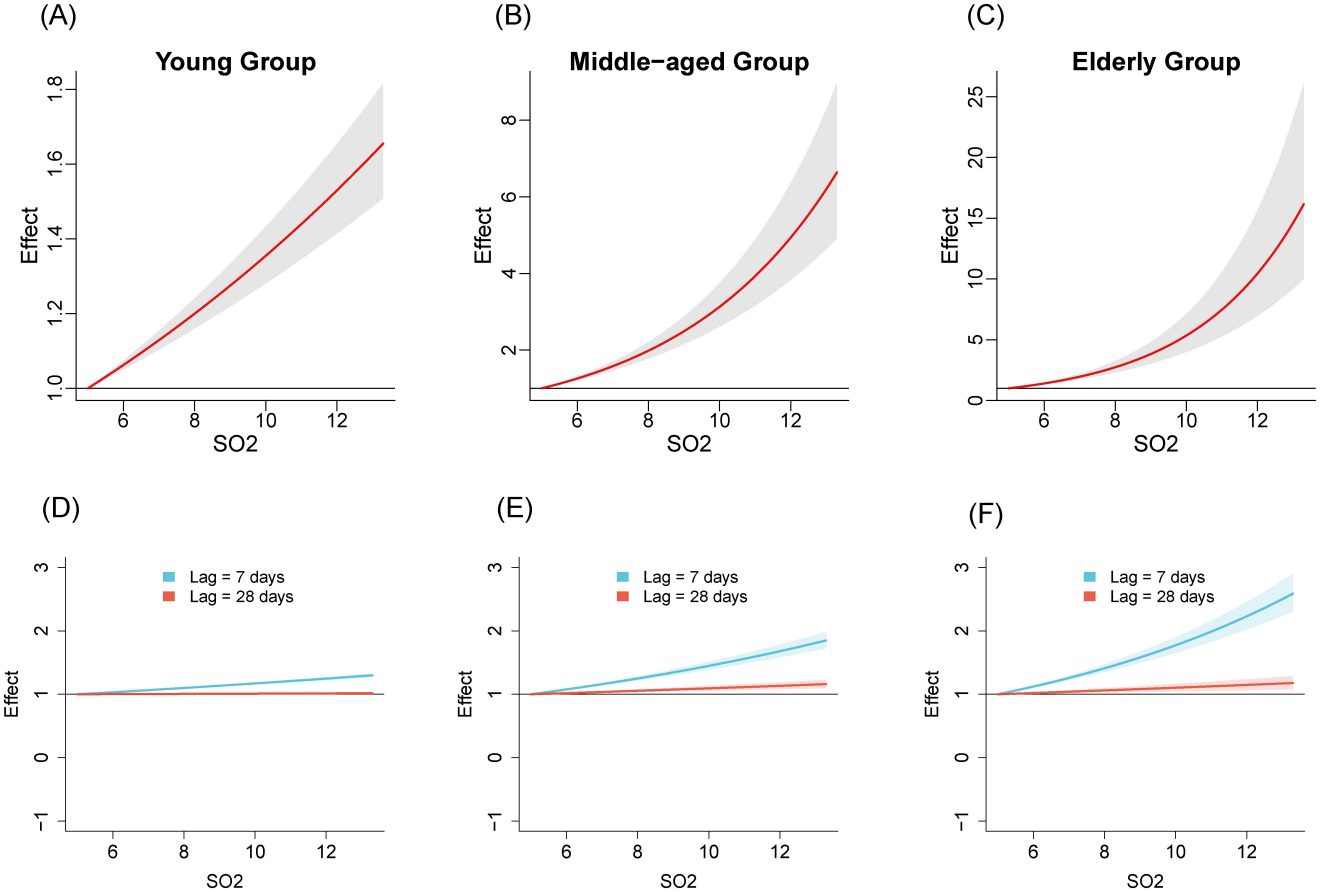
The estimated overall effects of SO_2_ (μg/m^3^) on influenza across 3 age groups using DLNM. Top row **(A–C)**: Cumulative effects of SO_2_ over the entire lag period, representing the overall impact on influenza risk. Bottom row **(D–F)**: Specific risk of influenza at lag periods of 7 days (**blue line**) and 28 days (**red line**). **(A, D)** Young group, **(B, E)** middle-aged group, **(C, F)** older adult group.

**Figure 3:**
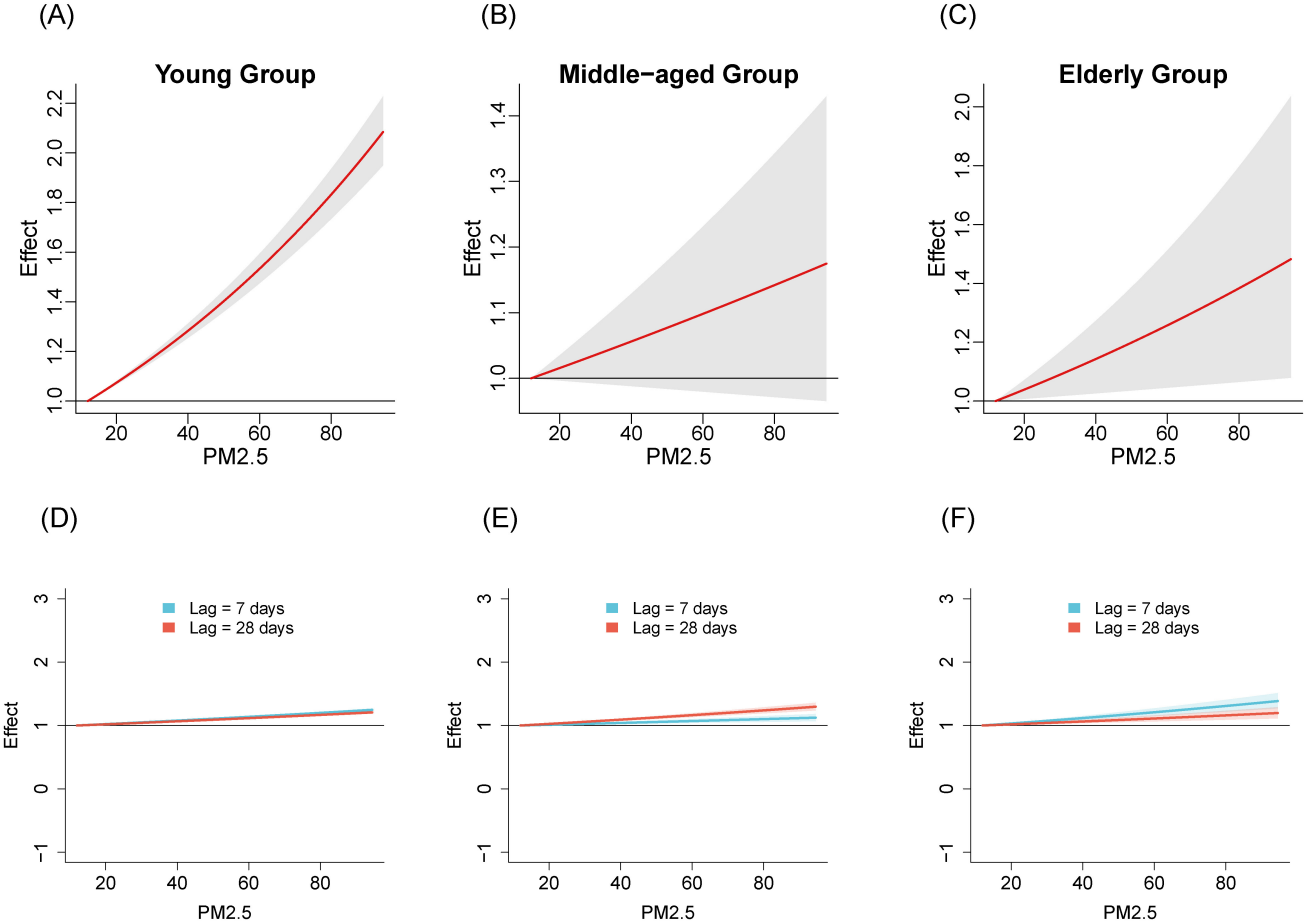
The estimated overall effects of PM_2.5_ (μg/m^3^) on influenza across 3 age groups using DLNM. Top row **(A–C)**: Cumulative effects of PM_2.5_ over the entire lag period, representing the overall impact on influenza risk. Bottom row **(D–F)**: Specific risk of influenza at lag periods of 7 days (**blue line**) and 28 days (**red line**). **(A, D)** Young group, **(B, E)** middle-aged group, **(C, F)** older adult group.

We compared the relationships between air pollutants and the number of influenza cases among different age groups from 2020 to April 2024. Figures 1–3 illustrate the association between air pollutants levels and the number of ILI cases across the three age groups. The upper panel presents the overall effect estimates, while the lower panel compares the effects at different lag periods (7 vs. 28 days). The exposure-response relationship between NO_2_ and SO_2_ and influenza shows a monotonous increasing trend across all age groups, while the relationship between PM_2.5_ and influenza shows a monotonous increasing trend in the young and older adult groups.

Table 2 shows the RR of influenza caused by extreme changes in pollutant concentrations, with the 1st percentile as the reference point. Compared to the reference concentration, when the pollutant concentration rises to the 99th percentile, the RR values quantifies the change in influenza risk associated with the sudden increase in pollutant concentration. In all age groups, increased concentrations of NO_2_ (RR = 5.02, 95% CI: 4.69–5.37; RR = 2.40, 95% CI: 1.97–2.94; RR = 3.17, 95% CI: 2.29–4.39 in the young adult, middle-aged adult, and older adult groups, respectively) and SO_2_ (RR = 1.47, 95% CI: 1.37–1.57; RR = 4.22, 95% CI: 3.36–5.30; RR = 8.31, 95% CI: 5.77–11.96, respectively) have both promoted the prevalence of influenza. The effect of NO_2_ on influenza is relatively greater in the young adult group, while the effect of SO_2_ is more pronounced in the middle-aged and older adult groups. Similarly, increased concentration of PM_2.5_ promoted the prevalence of influenza in the young group and older adult group (RR = 1.99, 95% CI: 1.87–2.12; RR = 1.45, 95% CI: 1.07–1.94, respectively). However, the effect of PM_2.5_ in the middle-aged group is not significant (RR = 1.16, 95% CI: 0.97–1.40).

**Table 2:** RR values for 99th vs. 1st percentile of pollutant concentrations.

**Age group**	**NO_2_**	**SO_2_**	**PM_2.5_**
Young	5.02 (4.69, 5.37)	1.47 (1.37, 1.57)	1.99 (1.87, 2.12)
Middle-aged	2.40 (1.97, 2.94)	4.22 (3.36, 5.30)	1.16 (0.97, 1.40)
Older adult	3.17 (2.29, 4.39)	8.31 (5.77, 11.96)	1.45 (1.07, 1.94)

Tables 3, 4 present the interaction effects between average temperature, relative humidity, and air pollutant concentrations, with all variables categorized based on their median values. Table 3 shows that, compared to high temperature and low pollutant concentrations, the relative risk (RR) increased under low temperature and high pollutant concentrations for NO_2_, SO_2_, and PM_2.5_, with RR = 1.59 (95% CI: 1.21–2.11), RR = 1.82 (95% CI: 1.36–2.44), and RR = 1.45 (95% CI: 1.10–1.89), respectively. Additionally, for SO_2_, the RR also increased under high temperature and high pollutant concentrations (RR = 1.60, 95% CI: 1.06–2.42). Table 4 indicates that, compared to high humidity and low pollutant concentrations, the RR increased under high humidity and high pollutant concentrations for NO_2_, SO_2_, and PM_2.5_ (RR = 1.59, 95% CI: 1.09–2.31; RR = 1.88, 95% CI: 1.30–2.71; RR = 1.60, 95% CI: 1.05–2.45, respectively). Moreover, NO_2_ and SO_2_ also exhibited increased RR under low humidity and high pollutant concentrations (RR = 1.44, 95% CI: 1.05–1.97; RR = 1.50, 95% CI: 1.12–2.01, respectively). To assess interaction effects, relative excess risk due to interaction (RERI), attributable proportion due to interaction (AP), and synergy index (SI) were employed. These metrics quantify the degree of interaction, estimate the proportion of excess risk attributable to interaction, and evaluate the synergistic effect between the two factors, respectively. However, since the 95% confidence intervals of RERI and AP included 0, and the 95% confidence interval of SI included 1, the interaction between temperature, relative humidity, and air pollutant concentrations did not reach statistical significance.

**Table 3:** Merged effects of average temperature (temp) and air pollutants on ILI cases count.

	**NO_2_**	**SO_2_**	**PM_2.5_**
	**RR (95%CI)**	**RR (95%CI)**	**RR (95%CI)**
High temp and low pollutant	Ref	Ref	Ref
Low temp and low pollutant	1.03 (0.59, 1.79)	1.12 (0.71, 1.78)	1.59 (0.74, 3.41)
High temp and high pollutant	1.25 (0.74, 2.12)	1.60 (1.06, 2.42)^*^	0.93 (0.31, 2.79)
Low temp and high pollutant	1.59 (1.21, 2.11)^*^	1.82 (1.36, 2.44)^*^	1.45 (1.10, 1.89)^*^
RERI (95%CI)	0.31 (−0.55, 1.17)	0.10 (−0.70, 0.90)	−0.07 (−1.65, 1.51)
AP (95%CI)	0.20 (−0.34, 0.73)	0.05 (−0.39, 0.50)	−0.05 (−1.14, 1.04)
SI (95%CI)	2.10 (0.10, 46.20)	1.14 (0.37, 3.48)	0.86 (0.04, 18.28)

The ^*^ indicates that the result is statistically significant.

**Table 4:** Merged effects of relative humidity (RH) and air pollutants on ILI cases.

	**NO_2_**	**SO_2_**	**PM_2.5_**
	**RR (95%CI)**	**RR (95%CI)**	**RR (95%CI)**
High RH and low pollutant	Ref	Ref	Ref
Low RH and low pollutant	0.89 (0.57, 1.39)	0.71 (0.42, 1.19)	0.82 (0.48, 1.41)
High RH and high pollutant	1.59 (1.09, 2.31)^*^	1.88 (1.30, 2.71)^*^	1.60 (1.05, 2.45)^*^
Low RH and high pollutant	1.44 (1.05, 1.97)^*^	1.50 (1.12, 2.01)^*^	1.28 (0.95, 1.72)
RERI (95%CI)	−0.04 (−0.72, 0.64)	−0.09 (−0.82, 0.65)	−0.15 (−0.94, 0.64)
AP (95%CI)	−0.03 (−0.50, 0.45)	−0.06 (−0.55, 0.43)	−0.12 (−0.73, 0.50)
SI (95%CI)	0.92 (0.22, 3.81)	0.85 (0.24, 2.99)	0.65 (0.10, 4.35)

The ^*^ indicates that the result is statistically significant.

## 4 Discussion

In this study, we collected the weekly reported influenza data in Jiangsu, China, during 2020 to April 2024. A total of 3,941,923 ILI cases were included and analyzed based on two stages of NPIs. Previous studies have shown that NPIs can partially alleviate seasonal influenza and potential pandemics in China (19, 20), and after the relaxation of certain NPIs, influenza activity increases significantly (21). Similar findings were obtained in the present study. As the COVID-19 spread, the public proactively chose to stay at home, resulting in a decrease in contact rate and a subsequent reduction in influenza cases. After the NPIs were relaxed, population mobility increased, leading to a rise in contact rate and influenza cases (22).

Considering the two stages of NPIs, we analyzed the exposure-response relationships for NO_2_, SO_2_, PM_2.5_ across the three age groups. The results revealed varying degrees of association between air pollutants and influenza prevalence in different age groups, suggesting age-specific effects of air pollutants on influenza transmission. NO_2_ shows a significant positive effect in all age groups, especially in the young group with the highest RR value, which is consistent with the conclusion drawn by Chen et al. (6). For the youth group, exposure to NO_2_ may lead to rapid release of immune cells, disrupting the balance of the immune system and making this age group more sensitive to influenza viruses (6, 23). The RR value of SO_2_ is significantly higher in the middle-aged and older adult groups compared to the young group, with a peak observed especially in the older adult group. This may be related to the chronic respiratory diseases commonly present in the older adult population, such as COPD (24). SO_2_ could potentially damage the immune response of the human respiratory system by triggering non-specific reactions in the airways. This may lead to impaired function of lung macrophages and a reduced rate of particulate matter clearance from the alveoli, making the body more susceptible to viral infections (25). The RR value of SO_2_ in the middle-aged group is also high. This might be because the middle-aged group is the main work group, who spend more time outdoors than the young and the older adult, thus increasing the incidence of exposure to air pollutants (26). For PM_2.5_, exposure to it affects the synthesis of pro-inflammatory cytokines in bronchial epithelial cell lines, disrupts antiviral signaling pathways, and reduces the production of antiviral cytokines (27–29). PM_2.5_ exhibits a positive effect in the young and older adult groups, while its effect in the middle-aged group is not significant. The impact of PM_2.5_ on different age groups may vary due to differences in immune system maturity, tolerance, and exposure duration (30, 31).

In this study, we analyzed the interaction between meteorological factors and air pollutant concentrations. However, based on the quantitative interaction indicators of RERI, AP, and SI, it was found that the interaction between meteorological factors and air pollutants was not significant.

Our study has several strengths. First, the study divides the entire period into two phases, corresponding to the implementation and relaxation of NPIs, which helps assess the impact of NPIs on the spread of influenza. Second, the population is categorized into three age groups, allowing for an in-depth investigation of how air pollutants affect the risk of influenza infection across different age groups. Third, the study examines the interactions between temperature, relative humidity, and air pollutant concentrations, providing a comprehensive understanding of how these factors collectively influence the spread of influenza.

However, several limitations in this study should be noted. First, our findings were not compared to those in parallel studies conducted in other provinces in China. In the future, more cross-dimensional and multiregional studies are required to consolidate the findings of this study. Second, laboratory testing data of influenza were not used; thus, our findings should be advanced to deeper analysis on influenza subtypes. Third, each subtype of influenza has unique transmission characteristics (32), and the predominant strains vary across regions during influenza epidemics (33). A key limitation of our study is the potential selection bias, as the data were collected from sentinel hospitals. Although these hospitals were chosen based on their ability to provide broad coverage of the local population, they may not fully represent the wider population due to their specific geographic and demographic focus.

To mitigate this, we made efforts to ensure that our data sample was diverse and the results could be generalized by including hospitals from different regions with varying demographic characteristics. However, this does not eliminate the potential for bias, and future research should include data from a wider range of healthcare settings to ensure that findings can be generalized to the broader population.

## Data Availability

The data analyzed in this study is subject to the following licenses/restrictions: The data is from a third party and the author has no right to disclose it. It can be shared after obtaining permission from the third party. Requests to access these datasets should be directed to Jiangsu Provincial Center for Disease Control and Prevention, Nanjing, PR China.
