# Supplementary material for: Exploration of the impact of air pollutants on the influenza epidemic after the emergence of COVID-19: based on Jiangsu Province, China (2020–2024)
Source: Front Public Health. 2025 Apr 14;13:1555430. doi: 10.3389/fpubh.2025.1555430 (PMC12034646; doi:10.3389/fpubh.2025.1555430)
Supplement: Supplementary file 1 [file Data_Sheet_1.docx]

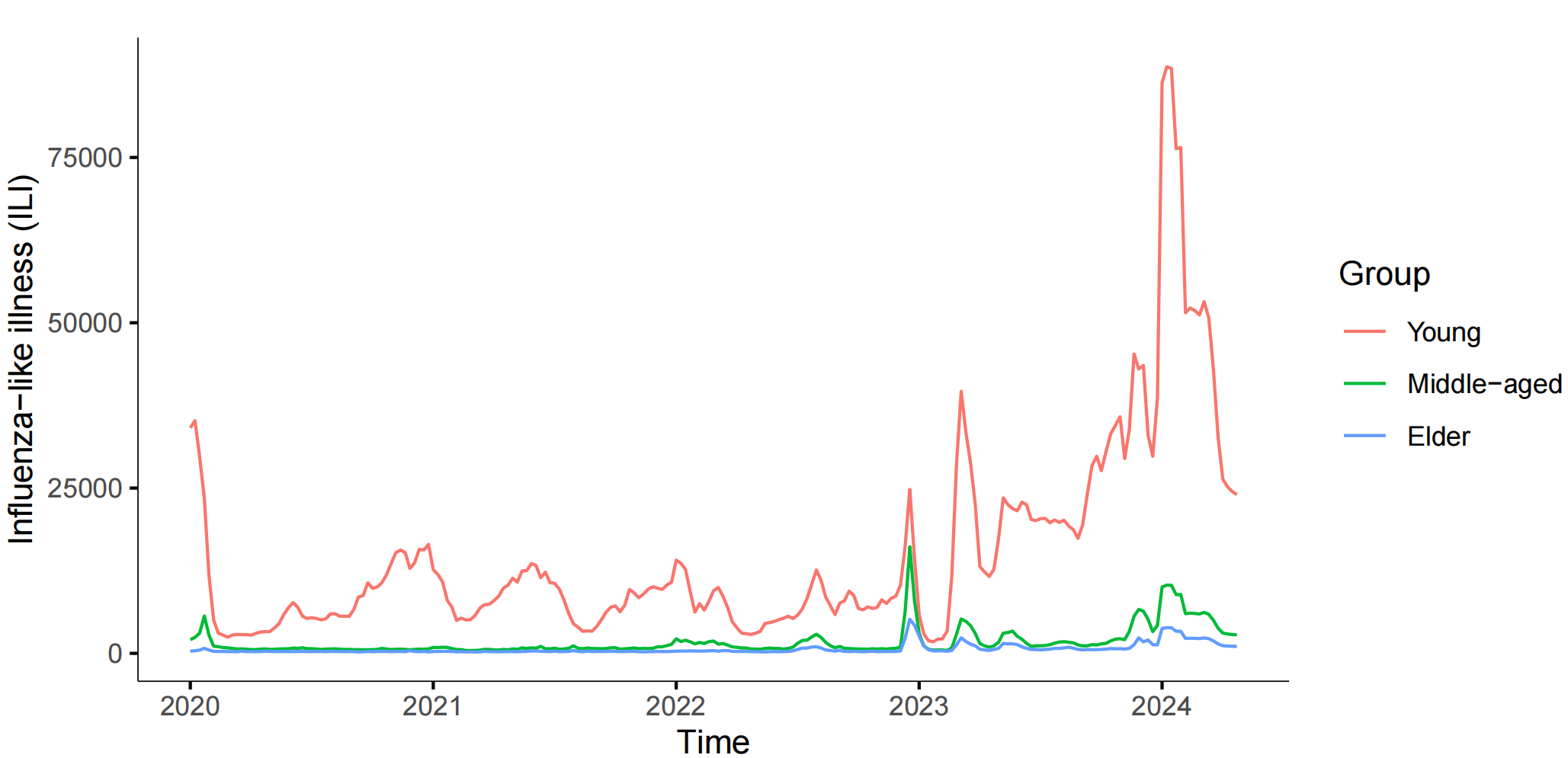


**Figure S1 ILI trends in young, middle-aged and elderly groups.**


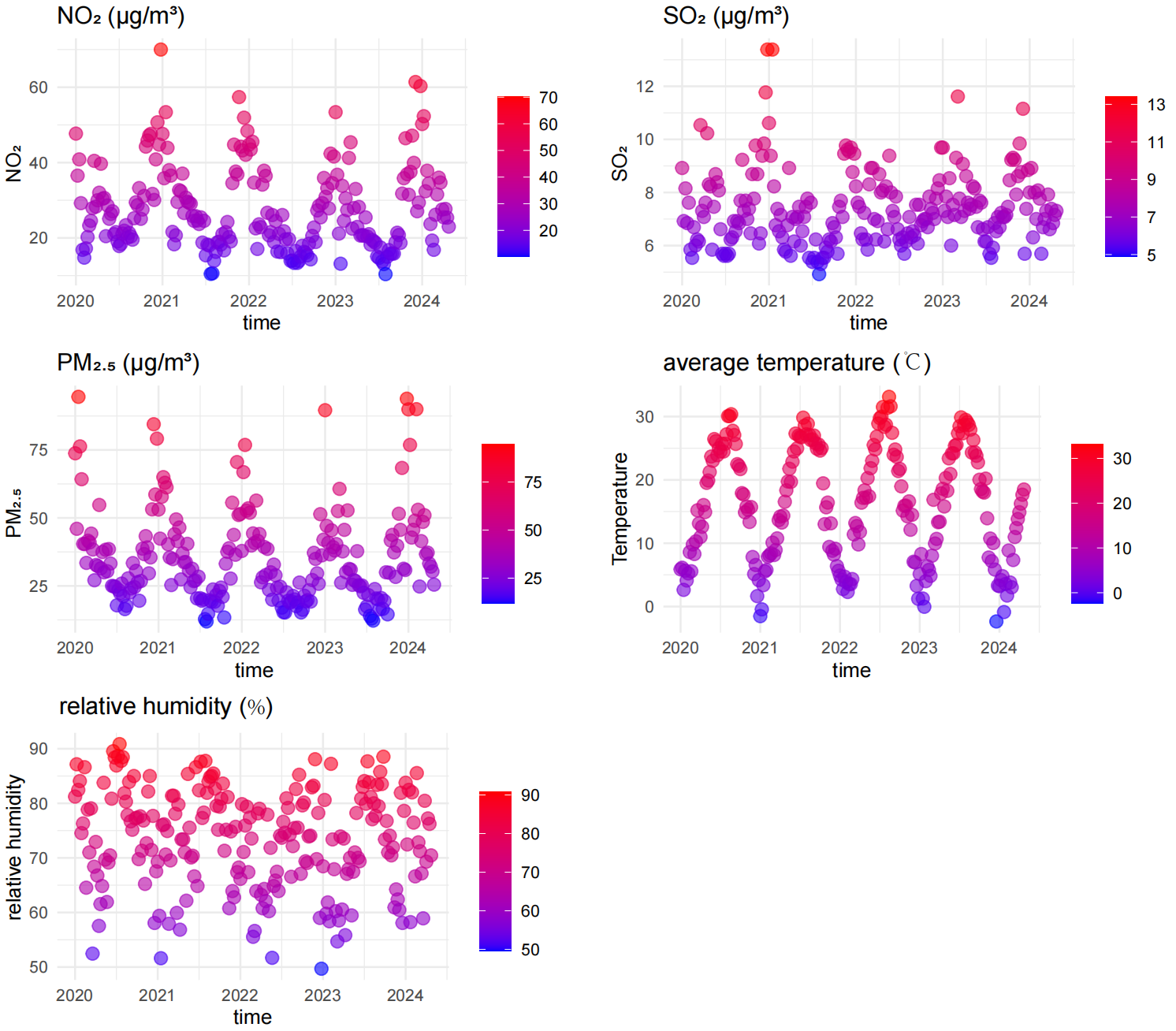


**Figure S2 Scatter plots of air pollutants and meteorological factors.**

**Table S1 The Akaike Information Criterion (AIC) values by changing the degrees of freedom (df) for “time”.**

|  | df | | | |
| --- | --- | --- | --- | --- |
|  | 4 | 5 | 6 | 7 |
| time | 33655.93 | 36569.76 | 25385.99 | 14535.80 |

**Table S2 The Akaike Information Criterion (AIC) values by changing the maximum lag weeks and degrees of freedom (df) for air pollutants.**

| Variables | Maximum lag (weeks) | df | | | |
| --- | --- | --- | --- | --- | --- |
|  |  | 1 | 2 | 3 | 4 |
| NO_2_ | 1 | 16180.63 | 16180.63 | 16180.63 | 16180.63 |
|  | 2 | 15438.12 | 15429.67 | 15429.67 | 15429.67 |
|  | 3 | 15705.10 | 15427.47 | 15212.79 | 15212.79 |
|  | 4 | 15237.81 | 14708.98 | 14709.91 | 14545.80 |
| SO_2_ | 1 | 17194.40 | 17194.40 | 17194.40 | 17194.40 |
|  | 2 | 16247.60 | 16201.34 | 16201.34 | 16201.34 |
|  | 3 | 16198.03 | 16196.81 | 16145.06 | 16145.06 |
|  | 4 | 16229.14 | 16128.52 | 15991.29 | 15973.50 |
| PM_2.5_ | 1 | 17102.44 | 17102.44 | 17102.44 | 17102.44 |
|  | 2 | 16556.93 | 16487.97 | 16487.97 | 16487.97 |
|  | 3 | 16514.95 | 16497.62 | 16373.99 | 16373.99 |
|  | 4 | 16042.58 | 15790.56 | 15687.81 | 15635.54 |
|  | | | | | |
